# Current Knowledge on the Preparation and Benefits of Cruciferous Vegetables as Relates to In Vitro, In Vivo, and Clinical Models of Inflammatory Bowel Disease

**DOI:** 10.1016/j.cdnut.2024.102160

**Published:** 2024-04-17

**Authors:** Tolu E Alaba, Johanna M Holman, Suzanne L Ishaq, Yanyan Li

**Affiliations:** 1 Graduate School of Biomedical Sciences and Engineering, University of Maine, Orono, ME, United States; 2 School of Food and Agriculture, University of Maine, Orono, ME, United States; 3 School of Pharmacy and Pharmaceutical Sciences, SUNY Binghamton University, Johnson City, NY, United States

**Keywords:** inflammatory bowel disease, cruciferous vegetables, oxidative stress, inflammation, microbiomes, glucosinolates, isothiocyanates, polyphenols and flavonoids

## Abstract

Inflammatory bowel disease is a chronic condition with a significant economic and social burden. The disease is complex and challenging to treat because it involves several pathologies, such as inflammation, oxidative stress, dysbiosis, and intestinal damage. The search for an effective treatment has identified cruciferous vegetables and their phytochemicals as potential management options for inflammatory bowel disease because they contain prebiotics, probiotics, and anti-inflammatory and antioxidant metabolites essential for a healthy gut. This critical narrative style review provides a robust insight into the pharmacological effects and benefits of crucifers and their documented bioactive compounds in in vitro and in vivo models, as well as clinical inflammatory bowel disease. The review highlights the significant impact of crucifer preparation and the presence of glucosinolates, isothiocyanates, flavonoids, and polyphenolic compounds, which are essential for the anti-inflammatory and antioxidative benefits of cruciferous vegetables, as well as their ability to promote the healthy microbial community and maintain the intestinal barrier. This review may serve as a viable nutritional guide for future research on methods and features essential to developing experiments, preventions, and treatments for inflammatory bowel disease. There is limited clinical information and future research may utilize current innovative tools, such as metabolomics, for adequate knowledge and effective translation into clinical therapy.

## Introduction

### Inflammatory bowel disease

Inflammatory bowel disease (IBD) is a chronic condition that can last a lifetime and reoccur, causing inflammation, diarrhea, rectal bleeding, and abdominal pain due to severe anatomical damage, immune overactivation, oxidative stress, and microbiome disruption [1,2]. Ulcerative colitis (UC) primarily affects the rectum and colon, leading to inflammation, pain, and diarrhea, including mucous and bloody stool, resulting in anemia and iron deficiency [1,3] as well as a greater risk factor for colon cancer, the second leading cause of cancer death globally [4,5]. Crohn’s disease (CD) involves chronic inflammation of the distal gut, leading to lesions along the entire intestine, immune intolerance of commensals, fever, tiredness, and complications such as stenosis, fistulas, and strictures [6]. Patients often experience remission and recurring flares resulting in surgical removal of damaged intestinal regions [7]. IBDs create a continuous pathological circle of comorbidities: malabsorption and malnutrition can lead to adipogenesis, obesity, diabetes, and nonalcoholic fatty liver disease [8,9]; IBD stress is associated with 8%–9% of pregnancy loss or preterm birth [10]; and it can impact psychological well-being, including anxiety, depression, obsessive disorder, and teasing from peers [11,12].

IBD is a significant economic and social burden, particularly in Europe and North America where rates are high [13,14]; in the United States, >3 million people suffer from IBD, with an annual cost of >$30 billion. Cases of IBD have dramatically increased in many countries [15], with various factors contributing, including diet and the switch to ultraprocessed foods [16], lifestyle, urbanization, exposure to environmental pollutants [17], and stress [18], as well as a few genetic markers [19,20]. Many studies suggest that diet is a major factor, including one that reported a 70% relationship between IBD and a switch to self-reported unhealthy diets with high sugar and saturated fats and less fiber, fruits, and vegetables [21].

IBD management focuses on alleviating inflammation, oxidative stress, or microbial dysbiosis and is challenged by severe side effects or individualized presentation of symptoms. Clinical medicine, such as immunosuppressants, antibiotics and steroids, and surgery [22,23], can induce remission, but many patients become less responsive over time. Patients can spontaneously enter remission, which masks medication’s loss of efficacy. Individuals can experience repetitive relapse [24], and patients with recurrent surgery often experience a high mortality rate [22,25]. Thus, a low-cost and efficient approach to alleviating oxidative stress, inflammation, and host–microbial dysbiosis is essential for IBD management [25,26]. This critical narrative review style highlights primary research articles documenting the preparation, use, and mechanisms of bioactive compounds derived from cruciferous vegetables as a potential management for IBD. There is evidence that a person’s response to plant fibers or secondary compounds can be highly individualized and reliant on their gut microbiota; thus, any ranking of the effectiveness of different brassicas would not be broadly applicable. This critical narrative style of review highlights that crucifer’s preparation affects the effectiveness and potential for targeting benefits in the gut.

### Comorbidities of oxidative stress and inflammation during IBD

The complex relationship between oxidative stress and inflammation could create a vicious cycle of chronic inflammation when immune cells produce reactive oxygen species (ROS) in response to intestinal injury, free radicals, and reactive nitrogen species [2] in the gut to recruit more inflammatory cells to exacerbate IBD [27,28]. Some critical pathways in the relationship between inflammation and oxidative stress are nuclear factor kappa B (NFkB) and nuclear factor erythroid 2-related factors 2/Kelch-like ECH-associated protein (NRF2/KEAP). The coupling of NRF2 and KEAP proteins within the intestinal cells could prevent the NRF’s transcription of antioxidants and anti-inflammatory proteins [29,30]. Meanwhile, NFkB could increase mitochondrial production of ROS by proton loss during intracellular enzymatic reactions, leading to uncontrolled oxidative stress, inflammation, and intestinal damage [31].

The gut epithelial barrier is critical to enforcing selective permeability to permit essential nutrients but prevent harmful substances [32]. Damaged barriers cause excess ROS, inadequate production of antioxidants, and leaky gut conditions [33] in which an influx of pathogenic bacteria activates NFkB epithelial and recruits immune cells, which disrupts gut microbiota homeostasis [34,35].

Similarly, the gut’s vasculature contains several tight junctions crucial for selective permeability and vascular dilation [36,37]. Mucosal endothelial cells can express vascular endothelial cell adhesion molecules (VCAM), intercellular-adhesion molecules (ICAMs), chemokines, and fractalkines to recruit and retain inflammatory cells [38]. Inflammatory signals, toll-like receptors (TLRs), and nucleotide oligomerization domains NOD1/2 via endothelial NFkB and mitogen-activated protein kinases could trigger sphingosine 1-phosphate receptor-2 for oxidative stress and further damage [39]. The endothelial-immune cell interaction within the gut could ultimately lead to platelet aggregation, intestinal coagulation, ischemia, and dysbiosis [39,40].

### Microbial dysbiosis during IBD

Microorganisms inhabit the human gastrointestinal tract, from the oral cavity to the rectum [41], colonizing the gut early in life through the parent’s microbiota and the living environment [42,43] and play an essential role in developing the child’s gut, cellular barrier, and mucosal layer [44,45], and tolerance to gut microbiota [46]. The composition of microbial communities varies by anatomical location and diet, which can either protect or predispose the gut to IBD [47,48]. Microbial populations in healthy people and patients with IBD are highly individualized, and changes associated with UC and CD are distinct. Moreover, there are differences between mucosal and luminal microbiota in inflamed and noninflamed tissues of patients with IBD [49].

Significant causal relationships exist between the gut immune system, oxidative stress, and loss of commensal bacteria [[50], [51], [52]]. Dysregulated inflammatory processes can cause inappropriate responses to regular gut commensals and lead to altered microbial architecture—dysbiosis. It is situationally specific whether dysbiosis precedes inflammation or dysbiosis leads to inflammation [53]. In UC, pathogenic microbiota can initiate IBD-like inflammation in germ-free mice [52,54], and some bacterial species of Clostridium, Escherichia coli, and Shigella flexneri can induce pathogenicity of commensals in the early development of IBD [55]. Similarly, in CD, pathogen infection and gut microenvironmental cues can cause the pathogenesis of commensal colonies to trigger dysbiosis [52]. Conversely, studies in mice showed that knockout of genes associated with host-microbe tolerance and increased copies of immune defense genes protected against gastrointestinal inflammation [56], indicating that inflammation can lead to dysbiosis.

Studies show that patients with IBD are likely to have lower microbial diversity [57], featuring more pathogenic bacteria and fewer beneficial species [58,59], which reduces short-chain fatty acid (SCFA) production but increases sulfur metabolism [60,61]. Therefore, dysbiosis is more pronounced in CD with increased pathogens such as Enterococcus spp., Lactobacillus fermentum, Clostridioides difficile, Shigella flexneri, and Listeria spp. and decreased commensals such as Faecalibacterium prauznitzii, Eubacterium rectale, Ruminococcus spp., Bacteroides fragilis, and B. vulgatus [47,62,63]. Meanwhile, reports from patients with UC showed increased bacterial phyla Pseudomonadota (formerly Proteobacteria), Actinobacteria, and Prevotella spp. and lowered Bacteroidota (formerly Bacteroidetes) [53]. Dysbiosis persists during disease inactivity, so decreased F. prausnitzii and Clostridium coccoides can predict relapse [64].

### The role of diet in IBD development and treatment

The development of dysbiosis, inflammation, and oxidative stress during IBD can be modulated by diet, ∼5 times more closely associated than genetic factors [2,[65], [66], [67]]. High consumption of dietary fat and sugar can increase mucosal dysbiosis, inflammation, and horizontal gene transfer in the microbiome to increase pathogenic colonization such as Pseudomonadota (formerly Proteobacteria) and Bacillota (formerly Firmicutes) phyla [68,69] and decrease SCFA-producing bacteria Roseburia spp., Eubacterium rectale, and Ruminococcus bromii [70,71]. Dysbiosis and damaged intestinal cells promote the influx of ROS and inflammasomes from impaired mitochondria biogenesis [72,73]: the hypoxic epithelial cells and inflammatory cytokines induce mitochondrial leaky electron transfer chain and peroxidase production for oxidative stress [74,75], which results in inadequate energy production and tiredness [75]. Mice fed with a high-fat and sugar diet exhibit severe edema, high leukocyte scores, and the presence of chemokines and cytokines, such as TNFɑ, IL-6, and chemokine (C-C) ligand2 (CCL2), in their colon [76], and fecal samples revealed more pathogenic bacteria, TLR expression, and neutrophil-to-lymphocyte ratio genes [76]. The same study also reported elevated concentrations of C-reactive proteins and monocytes in plasma of people consuming an ultraprocessed high-fat and high-sugar diet, indicating IBD-like high-fat–diet-induced systemic inflammation [76]. High-fat diets can reprogram innate immune cells via NOD-like receptor protein-3 signaling to promote systemic inflammation [77], and fat- and protein-rich diets triggered IL4, TNFɑ, and monocytes chemoattractant protein-1 and generated harmful peroxides [78,79]. Obesity is a significant link between type 2 diabetes and IBD severity with increased inflammation with decreased tight junction protein such as epithelial cadherin. These markers reflect epithelial permeability and inflammatory influx and retention within the colon [80]. Therefore, unhealthy diets promote dysbiosis, inflammation, and mitochondria-induced oxidative stress.

Meanwhile, healthy diets rich in fiber, antioxidants, and anti-inflammatory metabolites [81,82] can increase SCFA-producing bacteria to metabolize dietary micronutrients and phytochemicals for IBD treatment [[83], [84], [85]]. Vegetables with high content of glucosinolates and polyphenols can promote microbiota diversity [86,87], increase antioxidant activity, and reduce inflammation via increased dietary cysteine and microbiota production of SCFA [[88], [89], [90], [91], [92]]. SCFA can bind G protein-coupled receptors to alter inflammatory genes and increase the antioxidant activities of glutathione [[93], [94], [95], [96]]. Despite these benefits, there is a need for a comprehensive review of the preparation methods, models, and specific metabolic effects of cruciferous vegetables as a guide for translational research. Therefore, the discussion of this review will focus on the role of crucifers and their phytochemicals, which have bioactive properties against IBD development.

### Search criteria

The search criteria for this review include articles published between 2003 and 2023 in PubMed and Web of Science. We found 625 articles based on the keyword’s combination with inflammatory bowel disease, cruciferous vegetables, Brassicaceae, broccoli, inflammation, oxidative stress, microbiomes, microbiota, bioactive compounds, glucosinolates, isothiocyanates, polyphenols, flavonoids, gut barrier, and colon damage. Excluding criteria were cancer, microbial neuroinflammation, pharmacological or drug interventions in IBD, a disease associated with cruciferous plants, and noncruciferous sources of polyphenols and flavonoids, which reduced the searched articles to 220 publications. After checking for content relevance, we finally selected only 35 primary articles for the discussion section by excluding reviews, inadequate studies based on the reported information on extraction methods, and applications not associated with this review’s objectives.

## Discussion

### Cruciferous vegetables and bioactive compounds that mitigate IBD

Cruciferous vegetables belong to the mustard family and are rich in bioactive compounds beneficial to human health [97,98] via prebiotic, probiotic (i.e., when fermented), anti-inflammatory, and antioxidant effects for regulating gut microbiota communities and alleviating pathogenic signals of IBD [16,99,100]. They include broccoli and broccoli sprouts (Brassica oleracea variety italica), brussels sprouts (B. oleracea var. gemmifera), cabbage (B. oleracea var. capitata), cauliflower (B. oleracea var. botrytis), collard greens (B. oleracea var. viridis), kale (B. oleracea var. Acephala or B. napus), bok choy (Brassica rapa cultivar Chinensis), mustard greens (B. rapa and B. juncea), turnips (B. rapa, var. rapa), the mustard Virginia pepperweed/peppergrass (Lepidium virginicum), arugula (Eruca vesicaria sativa), and wasabi (Wasabia japonica, W. koreana, and Eutrema japonicum) [97,98]. The presence of glucosinolates (GSLs), flavonoids, and polyphenols in crucifers provide antioxidants to scavenge ROS and anti-inflammatory agents to alleviate IBD symptoms and heal intestinal damage [101,102].

**FIGURE 1:**
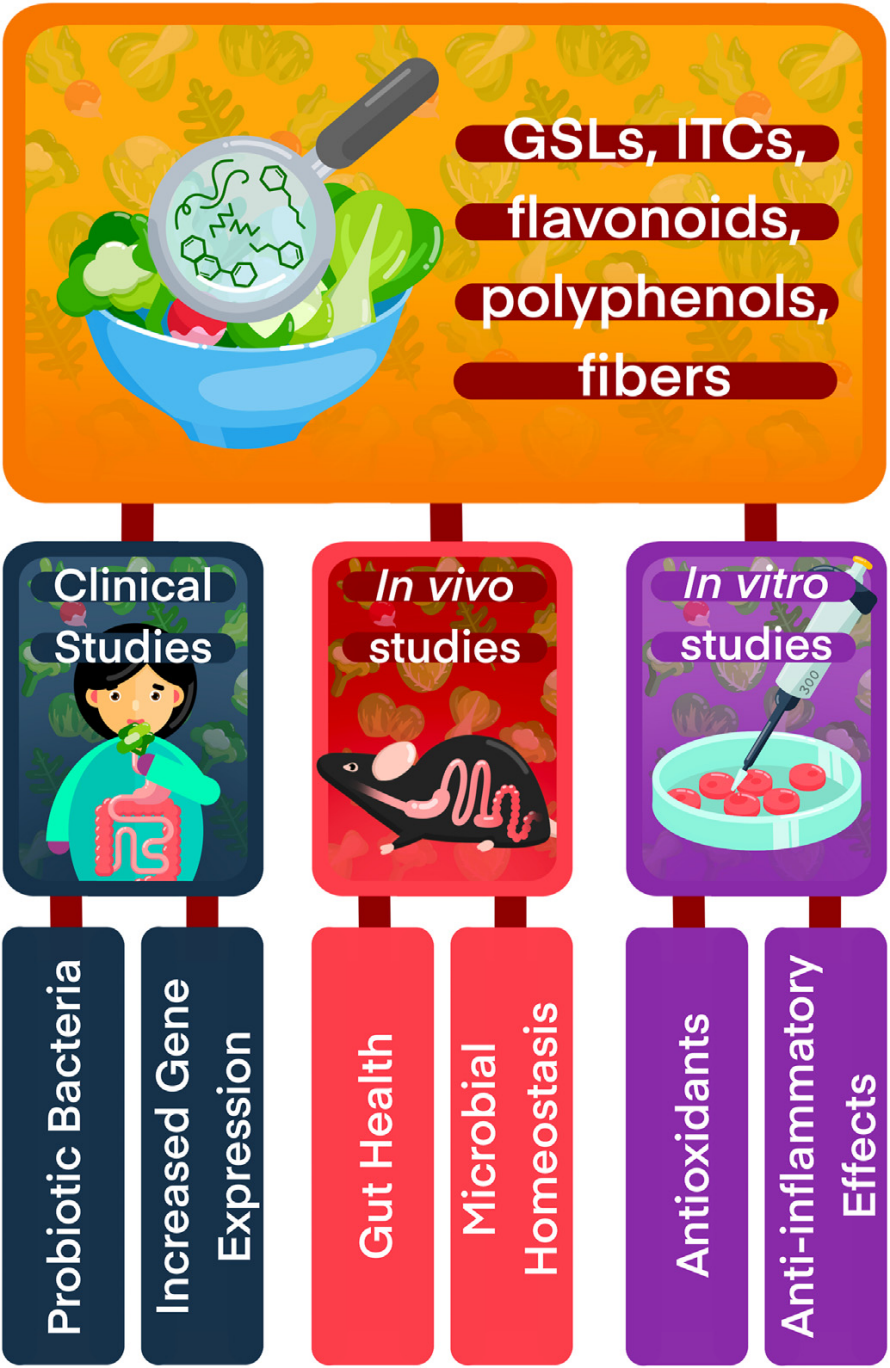
Inflammatory bowel disease is characterized by inflammation, oxidative stress, and dysbiosis, and clinical, in vivo, and in vitro studies have shown that dietary cruciferous vegetables can mediate these. Cruciferous vegetables and their phytochemicals, such as glucosinolates (GSLs), isothiocyanates (ITCs), flavonoids, and polyphenols, modulate inflammation, oxidative stress, dysbiosis, and gut barrier, as well as promote probiotic bacteria, microbial homeostasis, gut health, and upregulate anti-inflammatory and antioxidant genes. They represent a potential dietary management for patients with inflammatory bowel disease.

**FIGURE 2:**
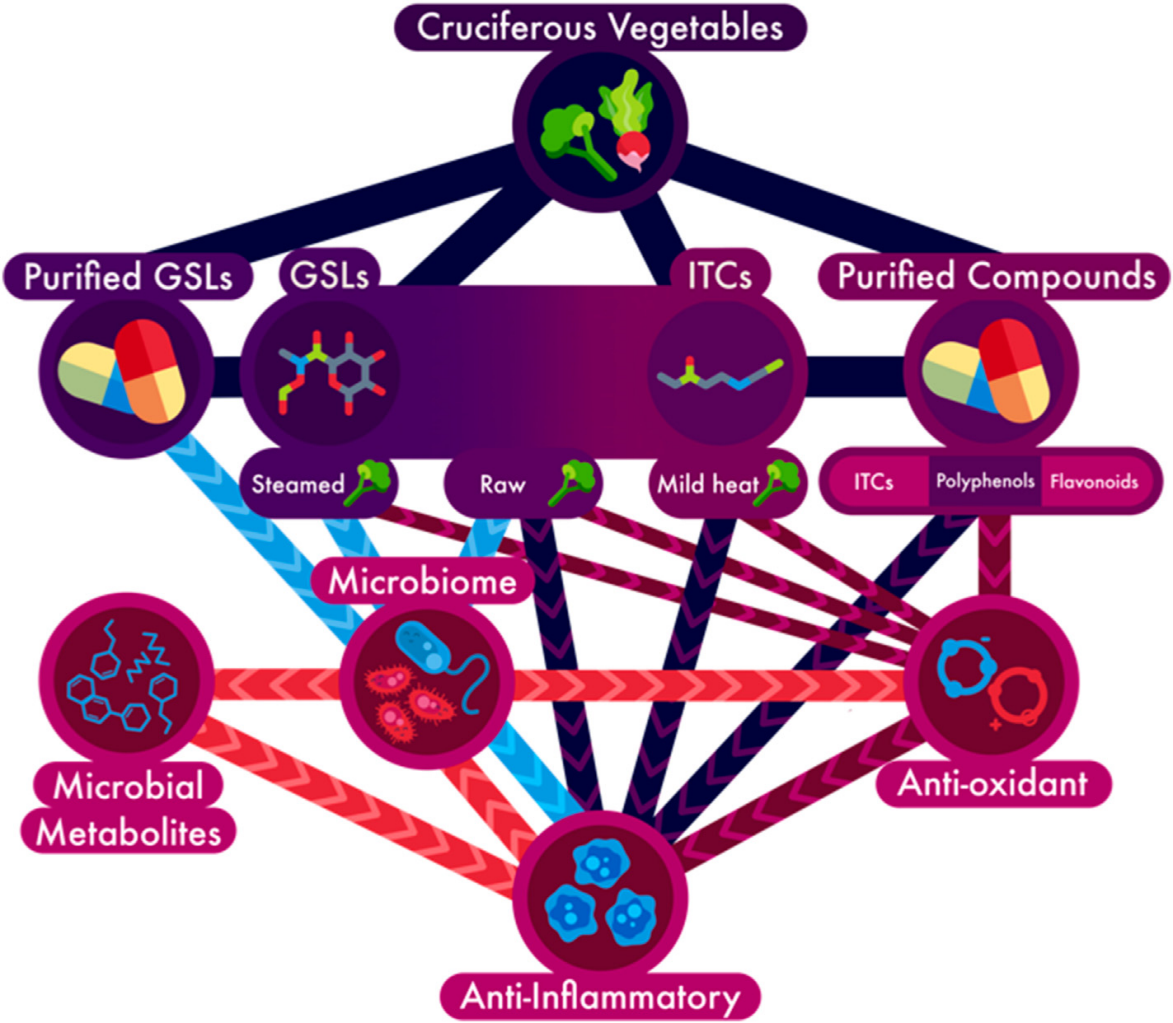
Cruciferous vegetables or their purified compounds can ameliorate inflammatory symptoms through multiple pathways. Fiber, glucosinolates (GSLs), isothiocyanates (ITCs), flavonoids, and polyphenols can reduce inflammation, immune activation, oxidative stress, and barrier damage and promote functional microbial communities. Each pathway has direct and indirect benefits.

Cruciferous diet can supply GSLs such as glucoraphanin (GLR), sinigrin, and glucoerucin, which are metabolized by plant-sourced myrosinase and gut microbiota-sourced enzymes into isothiocyanates (ITCs), such as sulforaphane (SFN) and erucin (Figure 1), for intestinal and systemic health benefits [[103], [104], [105]]. SFN is effective against IBD by maintaining Nrf2 redox homeostasis, protecting tight junctions, recruiting commensal bacteria, and increasing antioxidants and anti-inflammatory markers [30,106,107]. Broccoli sprouts possess more antioxidants, total phenolic content, and GSLs that provide ITCs than other crucifers [[108], [109], [110],^111^]. Meanwhile, broccoli, kale, radish, and cabbage are flavonoids-rich with quercetin and cyanidin, and polyphenols like ferulic, sinapic, and caffeic acids for prebiotic, endothelial and epithelial barrier protection, antioxidant, anti-inflammatory, and antiadhesive potential to complement the effect of ITCs against IBD [[110], [111], [112], [113], [114], [115]]. Importantly, cooking or other preparations of crucifers affect bioavailability of these phytochemicals (Figure 2) and where in the GI tract they will be absorbed. Future research should investigate the combined effect of phytochemicals from crucifers as a holistic approach to developing treatment or supplements for the clinical management of IBD.

### In vitro studies

#### Broccoli and broccoli sprouts

Broccoli has been widely studied for its anti-inflammatory and antioxidant properties against in vitro IBD models (Table 1 [[110], [111], [112], [113],[116], [117], [118], [119], [120], [121], [122], [123], [124], [125]] and Figure 1). One study showed that broccoli-derived nanoparticles (BDN), made of broccoli juice and lipids, prevented dendritic cell (DC) activation by upregulating AMPK anti-inflammatory pathways, thereby reducing chemoattractant molecules like chemokine (C-C) receptors (CCR), chemokine (CXC) ligand (CXCL), and CCLs in the DCs and monocytes [116]. Similarly, broccoli-derived vesicles rich in GLR, glucobrassicin, and neoglucobrassicin decreased oxidative stress [117] in Caco-2 cells (colon cells derived from a 72-y-old man with colorectal adenocarcinoma). The SFN treatment transformed the white blood cell monocytes (M1) to anti-inflammatory macrophages (M2) [118,119] through IL10 and signal-transducer and activator of transcription-3 pathways to ameliorate inflammation in bone marrow-derived macrophages [118], and decreased COX-2 and iNOS activities and increased IL10 and CD36 against chronic inflammation in human cells [119,126].

**TABLE 1:** Potential benefits of cruciferous vegetables in IBD in vitro models.

Cruciferous vegetables	Image	Bioactive compounds	Preparation	Dose	Treatmentperiod	Pathways	Benefits	Model	References
purified SFN	—	SFN	purified SFN	10,1 and 0.1 μmol/L	N.R.	IL10 activation of STAT3	Anti-inflammatory	Bone marrow-derived macrophages	[118]
Broccoli		SFN, lipids	Edible nanoparticles	N.R., mean diameter of 32.4 nm	2 and 3 h	CCRs, CXCLs, and CCLs	Anti-inflammatory	DCs and monocytes	[116]
		Glucoraphanin, Glucobrassicin, Neoglucobrassicin	Nano-vesicles	5 and 22 μg/mL	24 h	Hydrogen peroxide	Antioxidative	Caco-2 and NCI-H441 cells	[117]
		SFN	N.R.	100, 50, 20,10 and 1 μmol	24 h	COX-2 and iNOS, IL10 and CD36	Anti-inflammatory	Immortalized human blood monocytes	[119]
		Glucoraphanin, Glucoerucin, Quercetin-3-glucoside	Cold-pressed seed	0.1% (vol:vol)	0, 2, 4, 5, and 6 h time point	Bacteroidota/Bacillota ratio; ORAC, HOSC, and ABTS scavenging activities; COX-2 and IL1β cytokines	Antioxidant, Prebiotic, Anti-inflammatory	J774A.1 macrophages	[110]
Green cabbage and kimchi		Polyphenols	Freeze-dried	0.2, 0.5, 1 and 2 mg/mL	24 h	MCP-1, IL1β and TNFɑ, E. coli, S. aureus	Probiotic (kimchi), Anti-inflammatory	LPS- macrophages	[120]
Cauliflower		Polyphenols, Flavonoids	Ethanolic, hot and cold-water extract	1% and 50% (w/w)	24 h	DPPH, Lactobacilli	Antioxidant, Prebiotic	Lactobacilli strains	[121]
Kale		SFN, Beta Carotene	Ethanolic extract	100 μg/mL	28 h	IL6 and TNFɑ	Anti-inflammatory	LPS- macrophages	[113]
		Polyphenols: Ferulic and Caffeic acids	Frozen powder	100 μL	18 h	DPPH, E. coli and S. aureus suppression	Antioxidant, Antimicrobial	Microorganisms	[122]
		Total Polyphenol and Vitamin C	Freeze-dried powder and in vitro digestion	27 mg /100 g of polyphenols	18 h	Catalase, GSH, and SOD	Antioxidant	Caco-2, HT-29-MTX and THP-1 cells	[123]
Radish		Polyphenols	Chloroform, hexane, butanol, ethyl acetate, and water-soluble fractions	0, 1, 10, 100, and 200 μg/mL	48 h	PGE2, NFkB, COX-2 and iNOS, TNFɑ	Anti-inflammatory	LPS- macrophages	[124]
Radish Sprouts		Polyphenol: sinapic acid	Ethanol extract	10, 25, 50 and 100 μg/mL	4 h	NFkB, p-IkBɑ, IkBɑ, p65, iNOS, IL1β and TNFɑ	Anti-inflammatory	LPS- macrophages	[111]
Wasabi		Polyphenol: sinigrin acids	Ethanolic extract	100 and 500 μg/mL	18 h	NFkB, IL1β, TNFɑ, ZO-1, and occludin	Anti-inflammatory and barrier repair	Caco-2 cells and LPS- macrophages	[125]

This review suggests that the effects of cruciferous vegetables depend on the preparation and presence of bioactive compounds, which can alleviate inflammation, oxidative stress, dysbiosis, and gut barrier damage pathways during in vitro inflammatory bowel disease models.

ABTS, 2,2′-azino-bis(3-ethylbenzothiazoline-6-sulfonate); CCL, chemokine (C-C) ligand; CCR2, chemokine (C-C) Receptor; CD36, cluster of differentiation 36; COX-2, cyclooxygenase-2; CXCL, chemokine (CXC) ligand; DPPH, 2,2-diphenyl-1-picrylhydrazyl; DC, dendritic cells; GSH, glutathione; HOSC, hydroxyl radical scavenging capacity; HT-29-MTX, human colon adenocarcinoma cell line (ATCC HTB-38) resistant to 10^−6^ M methotrexate; IkBɑ, Inhibitor-kappa-B alpha; iNOS, Inducible nitric oxide synthase; J774A.1, mouse reticulum cell sarcoma; MCP-1, monocyte chemoattractant protein-1; NCI-H441, human epithelial adenocarcinoma; NFkB, Nuclear factor kappa B; N.R., data not reported in the article; RAC, oxygen radical absorbance capacity; PGE2, prostaglandin E2; p-IkBɑ, phospho-inhibitor-kappa-B alpha; SOD, superoxide dismutase; SFN, sulforaphane; STAT, signal-transducer and activator of transcription; THP-1, human acute monocytic leukemia cell line; ZO-1, zonula occludens-1.

Cold-pressed broccoli seed extracts (BSEs) exhibited prebiotic, anti-inflammatory, and antioxidant properties against LPS-induced inflammation in J774A.1 mouse cancer cells [110]. The treatment increased Bacteroidetes/Firmicutes ratio and production of SCFA and decreased commensal bacteria Akkermansia spp., Lactobacillus spp., and Bifidobacterium spp. [110]. BSE increased the antioxidant activities of ORAC, HOSC, and ABTS scavengers and inhibited COX-2 and IL1β cytokines above the control [110]. The benefits of BSE may be attributed to the presence of GLR, Glucoerucin, and quercetin-3-glucoside.

#### Other cruciferous vegetables

Culture media containing cabbage juice exhibited in vitro anti-inflammatory and prebiotic properties associated with indole-3-carbinol, a metabolite of glucobrassicin [115,120]. LPS RAW264.7 cells (macrophage-like, Abelson leukemia virus-transformed cell line derived from BALB/c mice) were cocultured with vegetable waste from green and kimchi cabbage. The kimchi inhibited 20% of IL6 activities more than green cabbage. However, both types of cabbage had similar inhibitory effects against LPS-induced nitric oxide inflammation. Furthermore, kimchi decreased E. coli significantly, whereas green cabbage had more effect against S. aureus [120].

The cold-water extract of cauliflower exhibited higher antioxidant activities of DPPH scavenger, whereas the ethanol extract possessed more flavonoids and polyphenols than both hot- and cold-water extracts [121]. Furthermore, all 3 extracts had prebiotic effects and promoted the growth of 6 Lactobacilli strains. Only hot- and cold-water extract significantly increased L. acidophilus microbial flora [121], which can promote a healthy gut.

One study identified 9 phenolic acids in kale, with ferulic and caffeic acids being the most abundant [113]. In the study, phenolic fractions of kale increased DPPH scavengers and decreased pathogenic bacteria, S. aureus, and E. coli [113]. An ethanolic extract from kale inhibited LPS-induced inflammation in RAW264.7 macrophages and relevant cytokines: IL6, iNOS, TNFɑ, and TLR-4 [122]. In a similar study, kale digesta rich in phenolic acids inhibited LPS and TNFɑ-induced intestinal epithelial cell inflammation and upregulated catalase, GSH, and superoxide dismutase antioxidant concentrations against cellular oxidative stress [123].

One study investigated different radish extracts with chloroform, hexane, butanol, ethyl acetate, and water-soluble fractions on LPS-induced inflammation in RAW264.7 macrophages [124]. The 100 μg/mL chloroform extract exhibited the most significant inhibition against NFkB, COX-2, iNOS, IL6, and TNFɑ concentrations [124]. Interestingly, the treatment also prevented platelet aggregation by decreasing cellular prostaglandin E-2 concentration [124]. Similarly, radish sprouts ethanolic extract (RSE) decreased IL6 and chemoattractant protein-1 concentrations in monocytes: the 100 μg/mL decreased IL1β and TNFɑ proteins the most, but both 50 and 100 μg/mL doses inhibited iNOS [111]. The RSE 100 μg/mL significantly inhibited NFkB protein by decreasing its association with inflammatory subunits, p-IkBɑ, and p65, while increasing the expression of the inhibitory subunit, IkBɑ [111].

Wasabi, a Japanese and Korean crucifer, exhibited anti-inflammatory properties and may repair intestinal barriers. Wasabi extracts cocultured with LPS-treated macrophages and Caco-2 cells inhibited NFkB, decreased IL1β and IFN-γ concentrations, and increased the expression of tight junction proteins zonula occludens-1 (ZO-1) and claudin, but not occludin, in Caco-2 cells [125]. In conclusion, these in vitro studies suggest the beneficial effects of cruciferous vegetables through prebiotic, anti-inflammatory, antioxidants, and gut barrier protective mechanisms, determined primarily by the crucifer’s preparation, bioactive compounds, dosage, and treatment period (Table 1 and FIGURE 1, FIGURE 2).

### In vivo studies

Similar to the findings from in vitro studies, broccoli has been reported to have anti-inflammatory, antioxidant, and prebiotic effects against IBD in animal models (Table 2 [48,99,102,105,107,111,112,116,118,122,125,[127], [128], [129], [130], [131], [132], [133], [134], [135], [136], [137], [138], [139], [140], [141]] and Figure 1). Both the UC and CD models use chemical triggers or genetic knockouts. The zymosan-mouse is a model of generalized gut inflammation using colon injection of zymosan to enhance the regeneration of immune-specific receptors for colitis and mucosal damage symptoms. Similarly, a generalized inflammation model is made using aryl hydrocarbon receptor (AHR), which is a ligand-activated transcription factor of the AHR with high ligand affinity or sensitivity in the Ahrb/b mice model (alleviates inflammation) and low ligand affinity or sensitivity in the Ahrd/d model (promotes inflammation).

**TABLE 2:** Potential benefits of cruciferous vegetables in IBD in vivo models.

Cruciferous vegetables	Image	Bioactive compounds	Preparation	Dose	Treatment period	Pathways	Benefits	Model	References
purified SFN	—	SFN	purified compound	10, 20, and 40 mg/kg	14 d	IL10 activation of STATs	Anti-inflammatory	DSS mice	[118]
purified SFN	—	SFN	purified compound	2.5, 5, 10 and 20 mg/kg	19 d	IL6 and TNFɑ, STAT3 and Nrf2, Bifidobacterium and Lactobacillus spp.	Prebiotic, Anti-inflammatory	DSS mice	[107]
Broccoli		SFN, GLR	purified	600 ppm	4 wk	IL1β and IL18, AMPK and PGC-1ɑ, Nrf2, HO-1, 8-OHdG	Anti-inflammatory, Antioxidant	DSS mice	[127]
		SFN	Raw and slightly cooked	10% (w/w)	2 wk	IL6, CCR2, and VCAM-1	Anti-inflammatory	DSS mice	[128]
		GLR, ITCs	Freeze-dried	4.1 mg/g	7 d	NQO1	Antioxidant	C57BL/6 mice	[99]
		SFN	Seed extract	370 mg/kg/d	2 wk	Bifidobacterium spp., Nrf2 signaling	Prebiotic, Anti-inflammatory	DSS mice	[129]
		Glucobrassicin	Freeze-dried	15% (w/w)	24 d	AHR, Cyp1a1, IL1β, IL10, IL6, Ptgs and CXCL5, increased Actinobacteria and Alistipes	Prebiotic, Anti-inflammatory	Ahrb/b and Ahrd/d mice	[130]
		GLR, SFN, and SFN-NAC	Raw seed extract	370 mg/kg/d	2 wk	IL1β, IL6 and TNFɑ, IL10, SOD, GSH, MDA, claudin-1, occludin and ZO-1, Bifidobacterium and Alistipes, SCFAs	Prebiotic, Anti-inflammatory, Antioxidant	DSS mice	[131]
		N.R.	Freeze-dried	10% (w/w)	21 wk	histologic injury score, E. coli, and Enterococcus spp.	Prebiotic, Anti-inflammatory	mdr1a−/− mice	[132]
		SFN	Crude extract	4%	12 d	NFkB, TNFɑ, IL10, MCP-1, and COX 2	No significance	DSS-rat	[102]
		N.R.	Juice	1.5 mL	7 d	IL8	Anti-inflammatory	DSS mice	[133]
		SFN, Lipids	Edible nanoparticles	mean diameter 32.4 nm	10 and 13 d	AMPK, IL10, TNFɑ, IL17A and IFN-γ	Anti-inflammatory	DSS mice	[116]
		SFN, Lipids	Edible nanoparticles	mean diameter 32.4 nm	10 d	TNFɑ, IL6, and IL23	Anti-inflammatory	Rag1−/− mice	[116]
Broccoli sprouts		GLR, SFN	Steamed and freeze-dried	10% (w/w)	34 d	Bacillota, Pseudomonadota, Bacteroidota and Verrucomicrobiota; TNFɑ, IL1β and IL6	Prebiotic, Anti-inflammatory	DSS mice	[48]
		SFN, GLR	Streamed	5% (w/w)	13 d	microbial hydrolase, Bacillota	Prebiotic	DSS mice	[105]
		SFN	raw	10% (w/w)	2 wk	E. coli and Helicobacter	Prebiotic, Anti-inflammatory	IL10-KO mice	[134]
Broccoli sprouts and seed		GLR, Glucoiberin, Glucoerucin, SFN-lysine	powder	100 mg	4 wk	Nrf2, NQO1, Gstm1, Srxn1, and GPx2	Anti-inflammatory and antioxidant	DSS mice	[137]
Brussels Sprouts		GLR	Cooked and freeze-dried	10% (w/w)	4 wk	Bifidobacterium and Lactobacillus	Prebiotic	human microbiota-associated rats	[135]
		GLR	cooked	10% (w/w)	4 wk	Β-glucuronidase, SCFA	Antioxidant, Prebiotic	human microbiota-associated rats	[136]
Bok Choy		Glucobrassicin, gluconapin, Progoitrin	powder	100 mg	4 wk	AHR, Cyp1a1, Nrf2, NQO1, Gstm1, Srxn1, and GPx2	Anti-inflammatory and antioxidant	DSS mice	[137]
Red Cabbage		Cyanidin 3,5-diglucoside	Freeze-dried powder	5% (w/w)	2 wk	IL6, IL1β, TNFɑ, iNOS, and COX-2	Anti-inflammatory	DSS mice	[138]
Kale		SFN, Beta Carotene	Freeze-dried powder	4.5% (w/w)	2 wk	NFkB, IL6, IL1β and TNFɑ, claudin-1 and occludin, Bacillota	Prebiotic, Anti-inflammatory	DSS mice	[122]
Radish		Polyphenol: sinapic acid	Water extract	10, 40, 70 and 100 mg/kg	7 d	GSH, MDA, IL1β, TNFɑ, iNOS, NFkB, MCP-1 and ICAM-1	Antioxidant, Anti-inflammatory	DSS and TNBS- Rat	[112]
Radish Sprouts		Polyphenol: sinapic acid	Ethanol extract	10, 50 and 100 mg/kg	14 d	NFkB, COX-2, iNOS, IL6, TNFɑ and PGE2, Bacteroidota, Pseudomonadota	Anti-inflammatory, prebiotic	DSS mice	[111]
Black Radish		ɑ-linolenic acid	Fermented	30 and 60 mg/kg	7 d	Histologic injury and colitis score	Anti-inflammatory	DSS mice	[139]
Virginia pepperweed		Polyphenols Flavonoids	Ethanolic extract	3, 30, and 100 mg/kg	4 d	MPO and CXCL2, IL1β and TNFɑ	Anti-inflammatory	DNBS- rat	[140]
Wasabi		Sinapic acid and ITCs	Ethanolic extract	100, 200 and 400 mg/kg	14 d	TNFɑ, Anxiety and pain test.	Anti-inflammatory	Zymosan- mice	[141]
		Polyphenol: sinigrin acids	Ethanolic extract	100 and 500 μg/mL	7 d	NFkB, IL1β, TNFɑ and ZO-1	Anti-inflammatory	DSS mice	[125]

This review suggests that the effects of cruciferous vegetables depend on the preparation and presence of bioactive compounds, which can alleviate inflammation, oxidative stress, dysbiosis, and gut barrier damage pathways during in vivo inflammatory bowel disease models.

Abbreviations: AHR, aryl hydrocarbon receptor; AMPK, 5' AMP-activated protein kinase; COX-2, cyclooxygenase-2; CXCL, chemokine (CXC) ligand; Cyp1a1, cytochrome P450 family 1 subfamily A member 1; DNBS, dinitrobenzene sulfonic acid; DSS, dextran sodium sulfate; GLR, glucoraphanin; GPx2, glutathione peroxidase 2; GSH, glutathione; Gstm1, glutathione S-transferase Mu 1; HO-1, heme oxygenase-1; ICAM-1, intercellular-adhesion molecule; IFN-γ, interferon-gamma; iNOS, inducible nitric oxide synthase; ITCs, isothiocyanates; MCP-1, monocyte chemoattractant protein-1; MDA, malondialdehyde; mdr1a, multidrug resistance protein 1a; MPO, myeloperoxidase; NRF2, nuclear factor erythroid 2-related factor 2; NAC, N-acetyl cysteine; NFkB, nuclear factor kappa B; NQO1, NAD(P)H quinone oxidoreductase 1; 8-OHdG, 8-hydroxydeoxyguanosine; PGC-1ɑ, peroxisome proliferator-activated receptor gamma coactivator 1-alpha; PGE2, prostaglandin E2; *Rag1*, recombination activating gene 1; SCFA, short-chain fatty acids; SOD, superoxide dismutase; SFN, sulforaphane; SFN-lys, methylsulfinyl ({[propyl]amino} carbonothioyl) lysine; Srxn1, sulfiredoxin1; STAT, signal-transducer and activator of transcription; TNBS, trinitrobenzene sulfonic acid; ZO-1, zonula occludens-1.

The DSS mouse or rat is the most commonly used UC model, with dextran sulfate sodium in drinking water used to chemically induce colitis with pathologies similar to human UC. The DNBS rat model uses intrarectal administration of dinitrobenzene sulfonic acid to induce diet- and stress-associated inflammation. *MDR1a*−/− mice are a UC model with genetically deleted multiple drug resistance genes to develop colitis spontaneously. *Rag1*−/− mice are a recombination activating gene1 knockout mouse model for chronic gut inflammation and fatal microbial infection. The *IL10*-knockout is the most popular immunological model for CD development. The TNBS rat model uses intrarectal administration of trinitrobenzene sulfonic acid to long-lasting pathologies and clinical symptoms of CD through the NOD2 pathway. In addition to these models to generate colitis, these mice and rats can be associated with human microbiota to assess gut microbial disorder.

#### Broccoli and broccoli sprouts

A study reported the effect of a broccoli diet rich in SFN and GLR against oxidative stress and inflammation in DSS mice. The diet decreased pathological score and TNFɑ, IL1β, and IL18 [127]. Meanwhile, it increased AMPK and PGC-1ɑ pathways and promoted Nrf2, HO-1, and 8-hydroxydeoxyguanosine (8-OHDG) activities against oxidative stress and DNA damage within the colon [127]. A similar anti-inflammatory effect was observed in DSS mice fed raw and lightly cooked broccoli diets [128]. Both diets decreased proinflammatory concentrations of IL6 and CCR2 but not TNFɑ in the colon, even though cooked broccoli had low SFN concentrations because myrosinase was inactivated, and thus, GLR conversion is reliant on gut bacteria. The diets improved mucosal regeneration and decreased neutrophil infiltration, disease activity index (DAI), and colonic damage scores. Interestingly, increased concentrations of tight junction proteins such as claudin-2, occludin, and ZO-1 and decreased VCAM-1 concentration may restore epithelial tight junction and risk of IBD flare [142]. Though this study confirmed that broccoli preparations can affect the immediacy of SFN bioavailability [142], cooking, which inactivates myrosinase and preserves GLR, can utilize gut microbiota to convert it to SFN directly in the colon [105] and other phytochemicals in any preparation could enhance the beneficial effects observed in cooked broccoli [128].

SFN from broccoli increased IL10 concentrations and inhibited IL6 and TNFɑ activities via signal-transducer and activator of transcription-3 and Nrf2 pathways in DSS mice [107,118]. The prebiotic effect of SFN treatment upregulated commensal bacteria such as Bifidobacterium and Lactobacillus bacteria in DSS mice [107], species that can metabolize GLR for SFN and SFN-N acetylcysteine bioavailability [129], and increased protective epithelial barrier proteins such as ZO-1, occludin, and claudin [143]. In another DSS mouse model, Bifidobacterium increased anti-inflammatory IL10 concentrations, inhibited TLR4, and decreased TNFɑ, IL1β, and IL8 cytokines in colon tissues. Bifidobacterium bifidum treatment decreased the pathogenic colonization of Streptococcus and Enterococcus and increased beneficial Actinobacteria to alleviate dysbiosis [143].

Broccoli is a rich source of AHR in the gut of mice that are sensitive to specific environmental toxicants and develop mucosal disorders and GI tumors, Ahrb/b, or the less sensitive, Ahrd/d mice [130]. Broccoli diet increased duodenal AHR-associated cytochrome P450, family 1, subfamily A, polypeptide 1 (Cyp1a1), and decreased IL1β, IL6, prostaglandin-endoperoxide synthase (Ptgs), CXCL5 concentrations and increased IL1O mainly in Ahrb/b mice to abrogate inflammation [130]. The broccoli diet was associated with beneficial bacteria, such as Actinobacteria and Alistipeds, and metabolic pathways, such as those involved in drug metabolism, valine/leucine/isoleucine biosynthesis, vitamins, and cofactors. At the same time, RNA seq analysis revealed *Reg1*, *Reg2*, *Tiff2*, and *CCL28* genes for gut barrier homeostasis, cell cycle regulation, cyclins, and checkpoints [130]. Thus, a broccoli diet may prevent barrier damage and DNA deregulation associated with intestinal stress.

BSE exhibited prebiotic, anti-inflammatory, antioxidant, and tight junction benefits in DSS mice. BSE promoted beneficial bacteria, such as species of Lactobacillus, Bifidobacterium, and Alistipeds, to increase SCFA production [131]. The BSE treatment decreased the oxidative concentration of malondialdehyde and increased the antioxidant concentrations of superoxide dismutase, and GSH inhibited inflammatory cytokines such as IL1β, IL6, and TNFɑ and increased anti-inflammatory IL10 [131]. The diet protected gut barriers by increasing claudin-1, occludin, and ZO-1 concentrations in the colon’s tight junctions [131]. Similar prebiotic and anti-inflammatory effects of freeze-dried broccoli decreased inflammation, histologic injury score, and pathogenic bacteria such as E. coli and Enterococcus in the *mdr1a*−/− mice model, which lack a multiple drug resistance gene and spontaneously develop colitis. A 10% broccoli diet induced beneficial cecal microbial communities, which increased SCFA richness, such as butyric and propionic acids [132].

Raw broccoli extract did not alleviate inflammation and oxidative stress in DSS mice [102], but interestingly, broccoli juice decreased serum IL8 concentrations to alleviate intestinal and systemic inflammation in DSS mice [133], again highlighting the importance of preparation’s effect on symptoms. Meanwhile, BDN increased AMPK to inhibit DCs and decreased mucosal inflammatory score. The colon concentration of IL10 increased, but TNFɑ, IL17A, and interferon-gamma (IFN-γ) were decreased in DSS mice. The study highlighted the role of SFN in activating AMPK and transforming DCs from immunogenic types to regulatory cells in the DSS and Rag1−/− mice [116].

Meanwhile, broccoli sprouts showed better effects against IBD due to higher GLR and SFN content. A steamed broccoli sprouts diet inhibited TNFɑ, IL1β, and IL6 and promoted bacteria richness such as Bacillota (formerly Firmicutes), Pseudomonadota (formerly Proteobacteria), Bacteroides, and Verrucomicrobiota against dysbiosis and IBD in DSS mice [48]. Microbial hydrolases and commensal microbiota promoted the beneficial effects of SFN [144] and SCFA production and decreased colonic ulceration [105]. Interestingly, eating broccoli sprouts early in life can improve microbiota richness. The study used IL10-knockout mice as a CD model and found higher serum SFN and microbial diversity in young mice than in adult mice. The diet decreased IBD symptoms, such as diarrhea, fecal blood, and pathobiont bacteria such as E. coli and Helicobacter, to alleviate dysbiosis and inflammation [134].

#### Other cruciferous vegetables.

Brussels sprouts exhibited prebiotic effects similar to broccoli sprouts, as reported in human microbiota-associated rats with improved gut microbiota diversity with increased commensal bacteria such as Bifidobacterium and Lactobacillus [135] and β-glucuronidase for SCFA production against intestinal toxicity and oxidative stress [136]. Meanwhile, broccoli sprouts and bok choy increased the expression of antioxidants such as Nrf2, NADH-Quinone oxidoreductase 1, Gstm1, Srxn1, and GPx2 to improve colitis symptoms [137].

Red cabbage alleviated colitis and intestinal inflammation by decreasing IL6, IL1β, TNFɑ, iNOS, and COX-2 concentrations in DSS mice [138]. The juice extracts increased microbiota diversity and Bacteroidetes-to-Firmicutes ratio due to the richness of cyanidin, a major flavanol from crucifiers [100]. Interestingly, Lactobacillus isolated from Chinese cabbage increased IL10 anti-inflammatory activity against colitis in DSS mice [145]. Kale exhibited similar anti-inflammatory and prebiotic effects in DSS mice. Freeze-dried powder of kale improved DAI scores and restored colon length. The diet also decreased inflammatory score, NFkB, IL6, IL1β, TNFɑ concentrations, and macrophage infiltration marker F4/80. Interestingly, kale promoted gut barrier integrity by increasing claudin-1 and occludin, decreased pathogenic Pseudomonadota (formerly Proteobacteria) such as E. coli and Enterobacter by 13%, and increased beneficial Bacillota (formerly Firmicutes) [122].

Radish has antioxidant, anti-inflammatory, and prebiotic benefits to alleviate IBD. Water extract of radish (RWE) reversed colitis and decreased the rats' body weight, colon length, DAI, and inflammatory damage scores. The diet suppressed inflammation and epithelial adhesion molecules by decreasing IL1β, TNFɑ, iNOS, NFkB, monocytes chemoattractant protein-1, and ICAM-1 concentrations in the colon. The RWE increased GSH and decreased malondialdehyde concentrations to attenuate oxidative stress [112]. Comparably, ethanol extract of radish decreased colon atrophy, inflammation, and prostaglandin E-2 platelet aggregation and improved microbiota diversity by increasing Bacteroidota (formerly Bacteroidetes) and Akkermansia spp. and decreasing Pseudomonadota (formerly Proteobacteria) such as E. coli and Enterobacter spp. The high content of sinapic acid, a polyphenol, was associated with the benefits of radish [111]. In contrast, the anti-inflammatory effect of fermented black radish was attributed to the presence of ɑ-linolenic acid and omega-6 acid [139].

Virginia pepperweed alleviated colitis and inflammation in the 2,4-dinitrobenzene sulfonic acid (DNBS) colitis rat. Its ethanolic extract was effective against IBD when administered intraperitoneally (i.p.) or orally. However, only the i.p. treatment completely restored weight loss and diarrhea and reversed intestinal damage by decreasing ulcers, edema, and inflammatory cells. Interestingly, both i.p and oral treatments alleviated bloody stool, whereas only the i.p decreased inflammatory concentrations of MPO, CXCL2, IL1β, and TNFɑ [140]. This study established the differential benefits of the 2 administration routes and the need to investigate further the various routes of cruciferous intervention in IBD studies.

A wasabi diet decreased colon inflammatory cells and serum TNFɑ concentrations to alleviate intestinal and systemic inflammation. Additionally, the diet reduced IBD-associated pain and anxiety behaviors in mice due to high sinapic acid content [141]. Similar effect was observed with sinigrin, a polyphenol from wasabi, inhibited NFkB and decreased inflammatory IL1β and TNFɑ concentrations. Interestingly, the compound also repaired the colonic barrier and increased the tight junction protein ZO-1 [125]. In conclusion, these in vivo studies suggest that cruciferous vegetables possess prebiotic, anti-inflammatory, antioxidant, and gut barrier protective benefits in animal models of IBD based on the preparation method, bioactive compounds, dosage, and treatment period (Table 2 and FIGURE 1, FIGURE 2).

### Clinical studies

Based on few clinical studies in the literature, we highlight the effects of various crucifer preparations on metabolites' bioavailability and potential antioxidant, anti-inflammatory, and prebiotic benefits, as shown in Table 3 [104,[146], [147], [148], [149], [150], [151], [152], [153]] and Figure 1.

**TABLE 3:** Potential benefits of Cruciferous vegetables in IBD clinical studies.

Cruciferous vegetables	Image	Bioactive compounds	Preparation	Dose	Treatment period	Pathways	Benefits	Subjects	References
BroccoMax supplement pill	—	SFN, ERN, GLR, Glucoerucin	Supplement	Six supplement pills	48 h	SFN, SFN-CG, and ERN-CG	SFN and ERN metabolites' bioavailability	Healthy participants	[147]
Broccoli		SFN, GLR	Fresh and Frozen	100 g	5 d	SFN, glutathione, SFN-CG, SFN-Cys, and SFN-NAC	SFN, ERN, and SFN conjugate bioavailability	Healthy participants	[104]
		SFN, SFN-NAC	cooked	200 g	24 h	SFN-NAC	SFN metabolites bioavailability	Healthy participants	[146]
		GSL metabolites	cooked	200 g	18 d	Bacteroidota/Bacillota ratio	Prebiotic	Healthy participants with high and low BMI	[149]
		GSL metabolites	cooked	200 g	17 d	SFN-Cys, SFN-NAC, SFN-GSH, ERN-Cys, ERN-GSH, ERN-NAC	SFN and ERN metabolite's bioavailability	Healthy participants with high and low BMI	[150]
		GSL metabolites	Frozen and steamed	300 g and 84 g	2 wk	Lactobacillus spp., microbial diversity	Prebiotic	Healthy participants	152
		N.R.	N.R., self-reported consumption	N.R., self-reported consumption	N.R., self-reported consumption	GPX3, IL23R, TNFɑ, and OCTN1/2	oxidative stress, inflammation, gut barrier	Patients with CD	[153]
Broccoli sprouts		SFN, ERN, GLR, Glucoerucin	Freeze-dried,	40 g	48 h	SFN-Cys, SFN-NAC, ERN-Cys, and ERN-NAC	SFN and ERN metabolites' bioavailability	Healthy participants	[147]
		SFN, SFN-Cys, SFN-CG, and SFN-NAC	Fresh or myrosinase-treated extract	200 μmol/d	24 h	P21, HDAC and HQ1	Antioxidant	Healthy participants	[148]
		SFN	cooked	45.7% (w/w)	14 d	Bacteroidota, Bacillota, Actinobacteria	Prebiotic	Healthy participants	[151]
Cabbage		SFN	raw	15.7% (w/w)	14 d	Bacteroidota, Bacillota, Actinobacteria	Prebiotic	Healthy participants	[151]
		N.R.	N.R., self-reported consumption	N.R., self-reported consumption	N.R., self-reported consumption	GPX3, GPX2, cadherin-29, ICAM1, STAT3, IL23R, IL12B, TLR9, and claudin-12	oxidative stress, inflammation, gut barrier	Patients with CD	[153]
Chinese cabbage		N.R.	N.R., self-reported consumption	N.R., self-reported consumption	N.R., self-reported consumption	IL23R and claudin-12	inflammation, gut barrier	Patients with CD	[153]
Cauliflower		SFN	cooked	36.4% (w/w)	14 d	Bacteroidota, Bacillota, Actinobacteria	Prebiotic	Healthy participants	[151]
		GSL	Frozen and steamed	84 g	2 wk	microbial diversity	Prebiotic	Healthy participants	[152]
		N.R.	N.R., self-reported consumption	N.R., self-reported consumption	N.R., self-reported consumption	GPX3, TNFSF15, cadherin-29, Janus kinase-2, NOD2 and OCTN1/2	oxidative stress, inflammation, gut barrier	Patients with CD	[153]
Horseradish		N.R.	N.R., self-reported consumption	N.R., self-reported consumption	N.R., self-reported consumption	OCTN1/2	gut barrier	Patients with CD	[153]
Mustard green		N.R.	Powder and sauce	N.R., self-reported consumption	N.R., self-reported consumption	IL6, ICAM1, NOD2, GPX3, GPX2, IL12B, NOD2, TNFSF15, and OCTN2	oxidative stress, inflammation, gut barrier	Patients with CD	[153]
Radish sprouts		SFN	raw	4% (w/w)	14 d	Bacteroidota, Bacillota, Actinobacteria	Prebiotic	Healthy participants	[151]
Rocket arugula		N.R.	N.R., self-reported consumption	N.R., self-reported consumption	N.R., self-reported consumption	IL6, ICAM1, GPX3, and claudin-2	oxidative stress, inflammation, gut barrier	Patients with CD	[153]

This review suggests that the effects of cruciferous vegetables depend on the preparation and presence of bioactive compounds, which can alleviate inflammation, oxidative stress, dysbiosis, and gut barrier damage pathways during clinical inflammatory bowel disease studies.

Abbreviations: CD, Crohn’s disease; Cys, cysteine; CG, cysteine-glycine; ERN, erucin; GLR, glucoraphanin; GSH, glutathione; GSL, glucosinolates; GPx, glutathione peroxidase; HDAC, histone deacetylases; HQ1, hydroquinone 1; ICAM-1, intercellular-adhesion molecule-1; NAC, N-acetyl cysteine; NAD(P)H quinone oxidoreductase 1; NOD2, nucleotide-binding oligomerization domain containing 2; N.R., data was not reported in the article; OCTN, organic cation/carnitine transporter; SFN, sulforaphane; SFN-lys, methylsulfinyl ({[propyl]amino} carbonothioyl) lysine; STAT, signal-transducer and activator of transcription; TLR9, toll-like receptor 9; TNFSF, TNF receptor superfamily.

#### Broccoli and broccoli sprouts.

In a randomized crossover clinical study, fresh and frozen broccoli soup had differential SFN bioavailability and metabolism. Healthy subjects who ate the fresh broccoli soup had 10 times more SFN in their plasma and urine than those who ate frozen diets [104]. Interestingly, the fresh broccoli soup had higher erucin and SFN conjugates in urine and fecal samples, which may be due to the gut microbial metabolism of GLR [104]. Similarly, participants who ate cooked broccoli with mustard seed powder had increased urine content of SFN and SFN-NAC 4 times more than broccoli alone [146] due to exogenous myrosinase from mustard powder.

A blinded crossover study on broccoli sprouts and supplements revealed differential SFN and ERN bioavailability (phase 1: 150 μmol glucoraphanin and 71 μmol glucoerucin; phase 2: 121 μmol glucoraphanin and 40 μmol glucoerucin) [147]. Participants who ate cooked sprouts showed maximum SFN and ERN concentrations in their plasma and urine after 3 and 6 h, respectively, whereas those who ate supplements had SFN and ERN peaks after 6 and 12 h [147]. Participants who ate sprouts had higher urine and plasma concentrations of SFN-Cys, SFN-NAC, ERN-Cys, and ERN-NAC. Meanwhile, supplements increased urine SFN/ERN ratio more than cooked sprouts, which may indicate more benefits [147]. Similarly, participants who ate myrosinase-treated broccoli sprouts had maximum urine and plasma SFN concentrations 3–6 h ahead of those who had cooked broccoli, again due to the immediate availability of SFN in raw or treated sprouts but the reliance on gut microbes to produce SFN in the colon using cooked sprouts. SFN-Cys and SFN-CG were majorly expressed at 3–6 h, although SFN-NAC was abundant after 12 h. However, 2 doses of sprouts diet at 12-h intervals had 3 times more SFN than a single dose per day. Meanwhile, broccoli sprouts and myrosinase-treated diets increased HDAC and HQ1 antioxidant concentrations against DNA oxidation at 2-dose consumption. Only the sprouts diet increased *p21* gene expression, which activates the Nrf2 antioxidant response. These studies revealed the importance of preparation, dose, and time in determining the benefits of broccoli against oxidative stress [148].

#### Combined effect of crucifers.

In a randomized crossover study, 10 healthy adults who consumed 200 g of cooked broccoli and 20 g of fresh winter radish for 17 d showed microbial diversity in their feces and metabolite diversity in their urine samples. The broccoli diet increased participants' urinal Bacteroidetes/Firmicutes ratio by 37% to promote gut diversity with increased GSL metabolites, especially in subjects with low BMI of <26 kg/m^2^, but the control diet decreased it by 5% [149]. Broccoli consumption also increased Bacteroides spp. by 8%, which is necessary for GSL microbial interconversion to ITCs [149]. A similar study reported a significant increase in plasma and urine metabolites of SFN and ERN after 4–18 h of consuming 200 g of cooked broccoli diet (providing 147.6 μmol of glucoraphanin and 3.6 μmol of glucoerucin) in individuals with high BMIs of >26 kg/m^2^ compared with individuals with low BMIs [150]. Another randomized crossover study examined the prebiotic effect of broccoli sprouts, radish sprouts, cauliflower, and cabbage as basal diets of low fruits and vegetables; a single diet of 7 g cruciferous vegetables; a double diet of 14 g cruciferous vegetables; a mixed diet of 7 g cruciferous and 4 g apiaceous vegetables [151]. The double cruciferous diets had higher SFN concentrations associated with beneficial gut microbiota such as Bacteroidota (formerly Bacteroidetes) to Bacillota (formerly Firmicutes) ratio and Actinobacteria in healthy subjects [151]. A similar study revealed a differential prebiotic effect of low (0.16 mmol GSLs) and high (2.7 mmol GSLs) broccoli and cauliflower diets. Participants who ate a high broccoli diet had microbial diversity with increased Lactobacillus spp., which may help protect against intestinal damage. In contrast, subjects who consumed low broccoli or only a cauliflower diet had no differential beta diversity [152], perhaps due to the low serving concentrations.

Consuming crucifers such as broccoli, cabbage, cauliflower, Chinese cabbage, arugula, watercress, horseradish, mustard sauce, and wasabi may have both beneficial and adverse effects in patients with CD, via the expression of genes associated with oxidative stress, inflammation, and gut barrier damage [153]. This study, conducted in New Zealand, reported interesting correlations between gene expression and self-reported adverse effects of crucifers, including broccoli diet and *GPX3*, *IL23R*, *TNFɑ*, and *OCTN1/2*; cabbage and *GPX2*, *GPX3*, *cadherin-29*, *ICAM1*, signal-transducer and activator of transcription-3, *IL23R*, *IL12B*, *TLR9*, and *claudin-12*; cauliflower and *GPX3*, *TNFSF15*, *cadherin-29,* *Janus* *kinase-2*, *nucleotide-binding oligomerization* *domain-2* and *OCTN1/2*; Chinese cabbage and *IL23R* and *claudin-12*; rocket arugula with *IL6*, *ICAM1*, *GPX3*, *and* *claudin-2*; watercress and *ICAM1* *and TNFSF15*; mustard greens and *IL6*, *ICAM1*, nucleotide-binding oligomerization domain-2, *GPX3*, *GPX2*, *IL12B*, *TNFSF15*, *and* *OCTN2*; horseradish and *OCTN1/2*; and wasabi correlated with *IL12B* and *OCTN2* [153]. For example, *GPX3*, a gene with protective effect against oxidative stress, had beneficial effects with broccoli and cauliflower diets but had an adverse effect with mustard sauce. Meanwhile, *NOD2*, a gene important for bacterial inflammatory response, had beneficial effects with mustard powder but had an adverse effect with cauliflower. Similarly, *CDH29*, a gene important for barrier junction organization, had a beneficial effect with cabbage but an adverse effect with cauliflower. This study revealed both positive and negative nutrigenomic effects of crucifers and the need for adequate experimental measures in cruciferous and IBD intervention. Further information, such as dietary data, lifestyle, specific dose, and preparation of the vegetables, could be more helpful in understanding the gene–diet relationship. In conclusion, the clinical studies suggest the importance of crucifers' preparation method, presence of bioactive compounds, dosage, and treatment period on their respective prebiotic, anti-inflammatory, and antioxidant benefits in healthy people and patients with CD (Table 3 and FIGURE 1, FIGURE 2).

### The role of metabolomics tools in cruciferous dietary IBD treatment

Metabolomics analysis is an effective tool for exploring dietary interactions with genetics, environment, gut microbiota, and signaling pathways [[154], [155], [156]]. Analyzing metabolite concentrations can identify pathophysiological pathways relevant to clinical IBD treatment [157]. A metabolomics profiling showed a positive correlation between dietary metabolites and the pathogenic expression of microbes: there were increased amino acids and Pseudomonadota (formerly Proteobacteria) spp. and decreased riboflavin and Faecalibacterium spp. in children with CD dysbiosis compared with children without dysbiosis [63].

Existing metabolomics have demonstrated the importance of dietary metabolites derived from cruciferous vegetables as modulators against IBD. For example, untargeted metabolomics revealed the differential expression of antioxidants and polyphenolic metabolites in raw and lactic acid bacteria (LABs)–fermented broccoli puree, using 2 strains of LABs, Lactiplantibacillus plantarum (LAB1) and Lactiplantibacillus pentosus (LAB10), relevant in phytochemical metabolism, which were isolated from fermented broccoli [158]. The LAB-fermented broccoli had more antioxidant activities than the unfermented broccoli after 60 h, with 20% increase in DPPH activities and 70% increase in FRAP and ABTS capacities [158]. Also, the LAB-fermented broccoli had abundant phenolic metabolites after 24 and 48 h, especially tryptophan and kaempferol [158]. Tryptophan alleviated clinical IBD symptoms of fatigue and inflammation [159], and kaempferol upregulated anti-inflammatory, antioxidant, and barrier protection markers [160]. This metabolomic analysis suggests that LAB fermentation may improve phenolics bioavailability and antioxidant benefits of broccoli intervention against IBD [158].

A comprehensive metabolomic and microbiota profiling revealed an important correlation between indole-3-carbinol, glucobrassicin metabolite, Roseburia spp., a butyrate-producing bacteria, and IL22, an anti-inflammatory cytokine, for gut protection against colitis [161]. Interestingly, decreased butyrate-producing bacteria, such as Faecalibacterium and Roseburia spp., have been identified in humans suffering from constipation [162]. Human fecal samples cocultured with digesta from broccoli and brussels sprouts revealed upregulated dietary metabolites. Metabolites, phenolic acids, and flavonoids, such as azelaic acid, sinapic acids, suberic acids, coumaric acids, kaempferol, flavonoid-O-3-glucosides, and indole acetic acids, showed positive relationships with both beneficial and detrimental microbiota [163]. Bifidobacterium spp. were increased, which helps GLR microbial transformation to SFN, whereas the Intestinibacter and Clostridiaceae genera were mostly unannotated [163].

It is essential to employ appropriate metabolomics analysis to efficiently identify metabolites with low sensitivity. For example, untargeted metabolomics identified the presence of glutathione metabolites in healthy human plasma samples, which was associated with the antioxidant benefits of the broccoli diet [164]. However, glutathione and its precursors, glutamine, and cysteine, were transiently downregulated at 6 and 12 h after the broccoli diet [165], whereas fatty acid metabolites associated with lipid metabolism were consistently downregulated [165]. There is a need for further investigation into this physiological effect of the broccoli diet against intestinal lipid peroxidation. Untargeted metabolomics did not reveal SFN and its metabolites in the urine of individuals who consumed 200 g of raw broccoli [166]. However, target metabolomics identified SFN, SFN-CYS, SFN-NAC, SFN-GSH, and other metabolites associated with SFN precursors mercapturic pathway in the same samples [166]. Similarly, a sensitive targeted LC/MS metabolomics method quantified plasma and urine SFN in humans after eating a 200 g broccoli diet. The authors synthesized SFN and mercapturic metabolites in the laboratory. They identified increased SFN, SFN-CYS, SFN-NAC, and SFN-GSH in specific quantities during intraday and interday plasma and urine measurements compared with control after the 4-wk broccoli diet [167]. Therefore, metabolomics profiling may be a significant tool to better understand the importance of dietary crucifers for gut health.

### Literature gaps, limitations, and perspectives for future work

The central gap in this literature review highlights the need for substantial publications on cruciferous vegetables’ translational and clinical effects on IBD management, as searching for clinical IBD studies generated articles primarily about immunosuppressant interventions and steroid therapies. Nutritional researchers may find it challenging to recruit IBD participants for studies if the medical institutions do not appreciate nutritional management of IBD with fibers such as cruciferous vegetables. Enlightening clinicians about the potential results of crucifers in IBD management by highlighting specific pathways of action would further encourage more participation in clinical studies. Proper documentation of cruciferous methods of preparation, extraction of biochemicals, dosage, application, and mechanisms of action in vitro and in vivo models will further encourage clinical studies and provide adequate information to the public and clinicians about the potential benefits of cruciferous consumption in IBD management.

## Conclusion

This review highlights cruciferous vegetables and phytochemicals with potential antioxidants, anti-inflammatory, and prebiotic effects that may prevent or alleviate IBD. Although many studies suggest that GSLs, ITCs, polyphenols, and flavonoids may benefit patients with IBD, there is a limited report on the mechanisms of polyphenols and flavonoids. Nonetheless, this information shows that flavonoids and phenolic compounds have beneficial properties that may work with GSL-derived SFN and conjugate to restore gut health and prevent IBD. The data in this review suggests broccoli and sprouts are the most widely studied crucifers with high bioactive compounds associated with the most reported IBD benefits. Future research should carefully consider diet preparation, dose, mode of administration, and intervention timeline as crucial factors that could impact crucifers' benefit in IBD research. Metabolomics is one of the significant tools currently being explored to identify and correlate the influence of dietary intervention on diseases. We recommend annexing metabolomics tools to investigate critical metabolites from cruciferous vegetables and their association with IBD pathways. This holistic approach may yield groundbreaking results for the clinical development of cruciferous vegetables as supplements and treatment recommendations for patients with IBD.

## Author contributions

The authors’ responsibilities were as follows – TEA: wrote the paper; JMH: contributed to writing the paper; SLI, YL: reviewed and edited the script; and all authors: have read and approved the final manuscript.

## Conflict of interests

The authors report no conflicts of interest.

## Funding

This project was supported by the USDA National Institute of Food and Agriculture through the Maine Agricultural & Forest Experiment Station: Hatch Project Numbers ME022102 and ME022329 (Ishaq) and ME022303 (Li); and the National Institute of Health [Li and Ishaq; NIH/NIDDK 1R15DK133826-01], and the Allen Foundation [Li and Ishaq, #5409406]. Financial sponsors had no role in study design, data interpretation, or report writing.
